# Genome mining of alkaliphilic cyanobacterial consortia: identification of biosynthetic gene clusters in *Sodalinema* and associated heterotrophs

**DOI:** 10.1128/aem.00780-26

**Published:** 2026-09-08

**Authors:** Ruchita Solanki, Everet Wiszniak, Lianchun Yi, Gamaliel Cabria, Marc Strous, Francisco Daniel Davila Aleman

**Affiliations:** 1 Department of Earth, Energy, and Environment, University of Calgary 2129, Calgary, Alberta, Canada; 2 Department of Biological Sciences, University of Calgary 2129, Calgary, Alberta, Canada; University of Minnesota Twin Cities, St. Paul, Minnesota, USA

**Keywords:** metatranscriptomics, metagenome assembled genomes, biosynthetic gene clusters, *Sodalinema*, cyanobacterial consortia

## Abstract

Alkaline soda lakes are high-pH environments that host specialized microbial communities with potential for biotechnology and natural product discovery. We characterized three *Sodalinema*-dominated cyanobacterial consortia enriched from Canadian soda lakes over 510 days. Using hybrid metagenomic sequencing and metatranscriptomics across pH, alkalinity, and temperature gradients, we reconstructed high-quality metagenome-assembled genomes and assessed functional activity. All consortia converged toward cyanobacteria dominance and exhibited temperature optima between 21°C and 30°C. Phylogenetic analysis placed *Sodalinema* genomes within a distinct clade affiliated with *Candidatus* Sodalinema alkaliphilum. Genomic analysis indicated complete biosynthetic pathways for vitamin B5, vitamin B7, and the molybdenum cofactor, but incomplete pathways for vitamins B1, B9, and B12, consistent with patterns observed in *Sodalinema yuhuli*. Metatranscriptomic profiles showed increased expression of genes involved in phycocyanin and carotenoid biosynthesis at pH 10.2 relative to pH 8.5. Biosynthetic gene cluster analysis revealed that most secondary metabolic potential resided in heterotrophic community members. *Roseinatronobacter* encoded pathways for N-acyl homoserine lactones, osmoprotectants, betalactones, and prodigiosin, while *Alkalimonas*, *Wenzhouxiangella*, and members of the *Kiloniellales* encoded clusters for lanthipeptides, cyclodipeptides, hydrogen cyanide, and pyrroloquinoline quinone. These findings indicate functional partitioning within the consortia and highlight the contribution of heterotrophs to secondary metabolism.

**IMPORTANCE:**

Alkaline soda lakes contain microbial communities adapted to high pH that remain underexplored for biotechnology. This study focuses on *Sodalinema*, a filamentous cyanobacterium that dominates enriched consortia from Canadian soda lakes, and its associated heterotrophic partners. We show that while *Sodalinema* drives primary productivity, heterotrophic bacteria encode most of the pathways for antimicrobial and signaling compounds. These interactions may support community stability and defense against competing microorganisms. By linking genomic potential with gene expression, this work identifies alkaline cyanobacterial consortia as a source of bioactive compounds and provides a framework for exploring extremophilic microbial communities for natural product discovery.

## INTRODUCTION

Cyanobacteria are highly adaptable microorganisms that thrive in a wide range of environments, from marine and freshwater to extreme niches, such as desert biocrusts, cave interiors, thermal springs, and alkaline soda lakes (1–4). Their ubiquity underscores their success as “primary producers,” as they perform photosynthesis to fix atmospheric carbon dioxide (CO_2_), produce oxygen, sustain global food chains, and offer a rich resource for bioproducts (5–7).

These adaptable traits allow the commercial success of cyanobacteria in the biotechnology industry—such as large-scale production of phycocyanin from *Limnospira platensis*. However, this industry faces significant economic and environmental challenges. For instance, phycocyanin production is inefficient due to the requirements of 800 L of water per kilogram and continuous CO_2_ sparging, which drives up costs (8–10). Moreover, as cyanobacteria consume bicarbonate, the rising medium’s pH converts the remaining dissolved inorganic carbon (DIC) into unusable carbonate (CO_3_^2−)^, forcing costly replacement of the growth medium. Cost reduction (>50%) can be achieved if CO_2_, nutrients, and water can be procured at a lower cost (11).

Cultivating cyanobacteria in media with both high pH (pH > 10) and high alkalinity (0.5 M), which mimic natural soda lakes, provides a unique and cost-effective solution. This environment can naturally “scrub” CO₂ from the atmosphere to regenerate usable DIC forms (HCO₃⁻ and CO_2_), reducing CO_2_ supply costs (12). The extreme pH also reduces the risk of contamination and system crashes by inhibiting the growth of competing microbes (13, 14).

Reliance on laboratory-grown “monocultures” like *Limnospira platensis* presents another challenge, as they are susceptible to abiotic stress, viral attacks, and microbial invaders (15, 16). Microbial ecology suggests that species diversity can promote ecosystem productivity and stability (17). Consequently, using designed or naturally occurring cyanobacterial consortia has the potential to enhance system stability (18, 19).

*Sodalinema*, a filamentous cyanobacterium, is an abundant member of microbial communities in alkaline soda lakes and represents a promising candidate for sustainable biotechnology (20, 21). Its inherent tendency to form synergistic consortia is a key advantage, as interspecies nutrient exchange enhances both metabolic efficiency and community resilience (22). Researchers successfully cultivated a *Candidatus* Sodalinema alkaliphilum consortium, enriched from Canadian alkaline soda lakes, in both outdoor photobioreactor and open raceway pond settings (23–25).

Cyanobacteria are known to produce a diverse range of compounds—including polyketides, peptides, and terpenoids—that possess valuable bioactivities such as antibacterial, antifungal, and antitumor properties (26–28). However, most known pharmaceutical candidates originate from marine or freshwater species, and little is known about the metabolites produced by alkaliphiles. The extreme conditions of soda lakes select for unique metabolic strategies, driving the evolution of specialized biosynthetic pathways and novel secondary metabolites (29, 30). Genome mining approaches, which systematically identify genes involved in secondary metabolite biosynthesis, have revealed an unexpectedly high biosynthetic capacity across diverse microbial lineages, underscoring the potential of understudied taxa (31–34). In this context, comprehensive genomic characterization of the *Sodalinema* consortia could provide the foundation for the discovery of previously unrecognized secondary metabolites.

The objective of this study was to characterize distinct cyanobacterial consortia of *Sodalinema* species from Canadian alkaline soda lakes via cultivation and whole-genome sequencing. The consortia were enriched at different temperatures, pHs, and alkalinities. Long-read (Nanopore) and short-read (Illumina) sequencing data were combined to generate complete, high-quality genomes. High-quality metagenome-assembled genomes (MAGs) of *Sodalinema* strains and their associated heterotrophs provided insight into each consortium’s taxonomic composition and metabolic potential. Specifically, biosynthetic gene clusters (BGCs) were identified, and metabolic pathways were reconstructed. In addition, metatranscriptomic analysis explored the responses of the consortia to different growth conditions.

## RESULTS

### Selective enrichment and eco-physiology

Fresh microbial mats were collected in early spring from three distinct soda lakes within the Cariboo Plateau: Deer Lake (DL), Probe Lake (PL), and Goodenough Lake (GE). Following collection, the mats were subjected to targeted enrichment to cultivate metabolically distinct microbial consortia. The DL and PL mats were initially cultured in a medium of moderate pH and alkalinity (8.5 and 0.3 M, respectively, LA3) (Table 1). Conversely, the GE mat was cultivated under significantly more alkaline and high pH conditions: pH 10.2 and 0.5 M alkalinity (HA3, Table 1). Incubation temperatures also differed among enrichments: DL and GE mats were cultivated at room temperature (21°C), whereas the PL mat was cultivated at 30°C. These temperatures were selected as permissive enrichment conditions rather than direct simulations of lake-specific *in situ* temperatures. Together with variation in medium pH and alkalinity, this design was intended to broaden the range of cyanobacteria and associated heterotrophs recovered from the mat samples.

**Fig 1:**
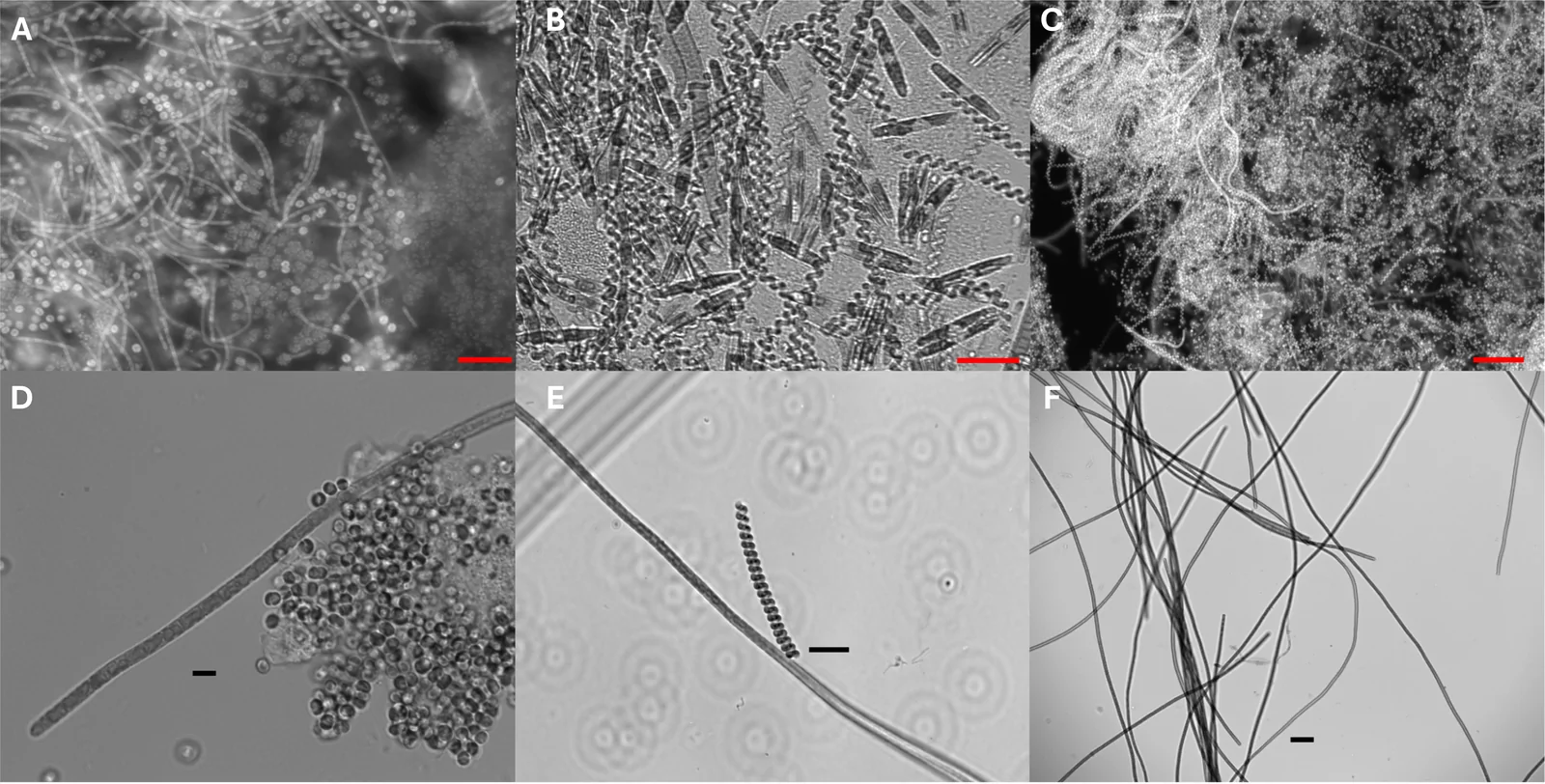
Bright-field and fluorescent micrographs of initial microbial mats (**A–C**) and bright-field micrographs of cyanobacteria enriched in three consortia: (**D**) Deer Lake (DL), (**E**) Probe Lake (PL), and (**F**) Goodenough (GE). Scale bar = 10 µm.

After 1 year of cultivation with regular medium exchange, all three consortia were dominated by filamentous cyanobacteria based on microscopy (Fig. 1). Cultures were maintained by replacing half of the spent medium with fresh medium every 15 days, as described in Materials and Methods. The dominant cyanobacteria occurred as cylindrical, unbranched filaments, consistent with *Sodalinema*, a genus previously enriched from the same soda lakes in photobioreactors (19, 35).

**Fig 2:**
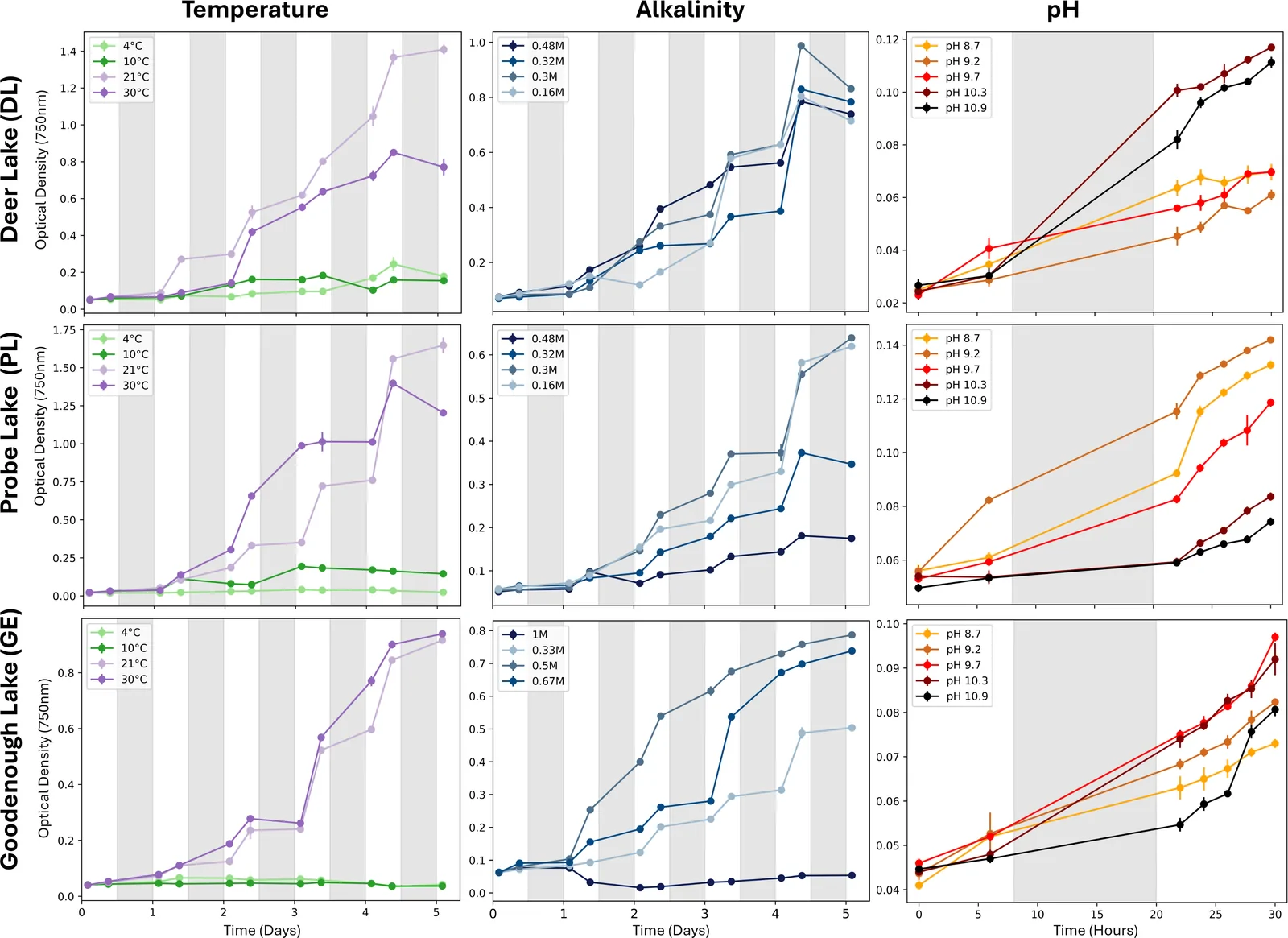
Growth dynamics of enriched microbial consortia under varying temperature, alkalinity, and pH conditions. Optical density (OD₇₅₀) measurements are shown for consortia originating from Deer Lake (DL) (top row), Probe Lake (PL) (middle row), and Goodenough Lake (GE) (bottom row). Left panels depict growth across temperature treatments; middle panels show growth responses to increasing alkalinity (HA1-4a and LA1-4b); right panels show growth at different pHs (pH 1–4a,b). Duration of the latter was shorter to limit the pH change associated with growth. Shaded gray regions indicate dark periods.

After a year of growth, these consortia were subjected to growth experiments at different pH, alkalinity, and temperature, to determine their optimum growth conditions. Across alkaline soda lakes in the Cariboo Plateau, water temperature varies seasonally from below freezing in winter to approximately 20°C in summer, while reported pH and alkalinity range from 9.0 to 10.7 and 0.2 to 2.0 M, respectively (21, 36). These values reflect regional and seasonal variation across lakes, rather than the full range occurring within each individual lake. In this context, we investigated how growth of the enriched consortia depended on environmental conditions (Fig. 2).

All three communities exhibited mesophilic growth with regard to temperature, with minimal to no growth observed at cooler temperatures (4°C and 10°C) and robust growth at warmer temperatures (21°C and 30°C). Specifically, the Deer and Probe Lake consortia showed fastest growth at 21°C. Despite the higher temperature (30°C) during one year of enrichment of the Probe Lake consortium, it did not have a higher optimum temperature. In contrast, the Goodenough Lake (GE) community grew well at both 21°C and 30°C, indicating a broader temperature optimum.

Further, the GE consortium (enriched at high alkalinity/pH) and the DL consortium (enriched at lower alkalinity/pH) had similar alkalinity and pH optima. Apparently, the applied differences in temperature, pH, and alkalinity did not drive selection during enrichment.

### Microbial community dynamics during enrichment

**Fig 3:**
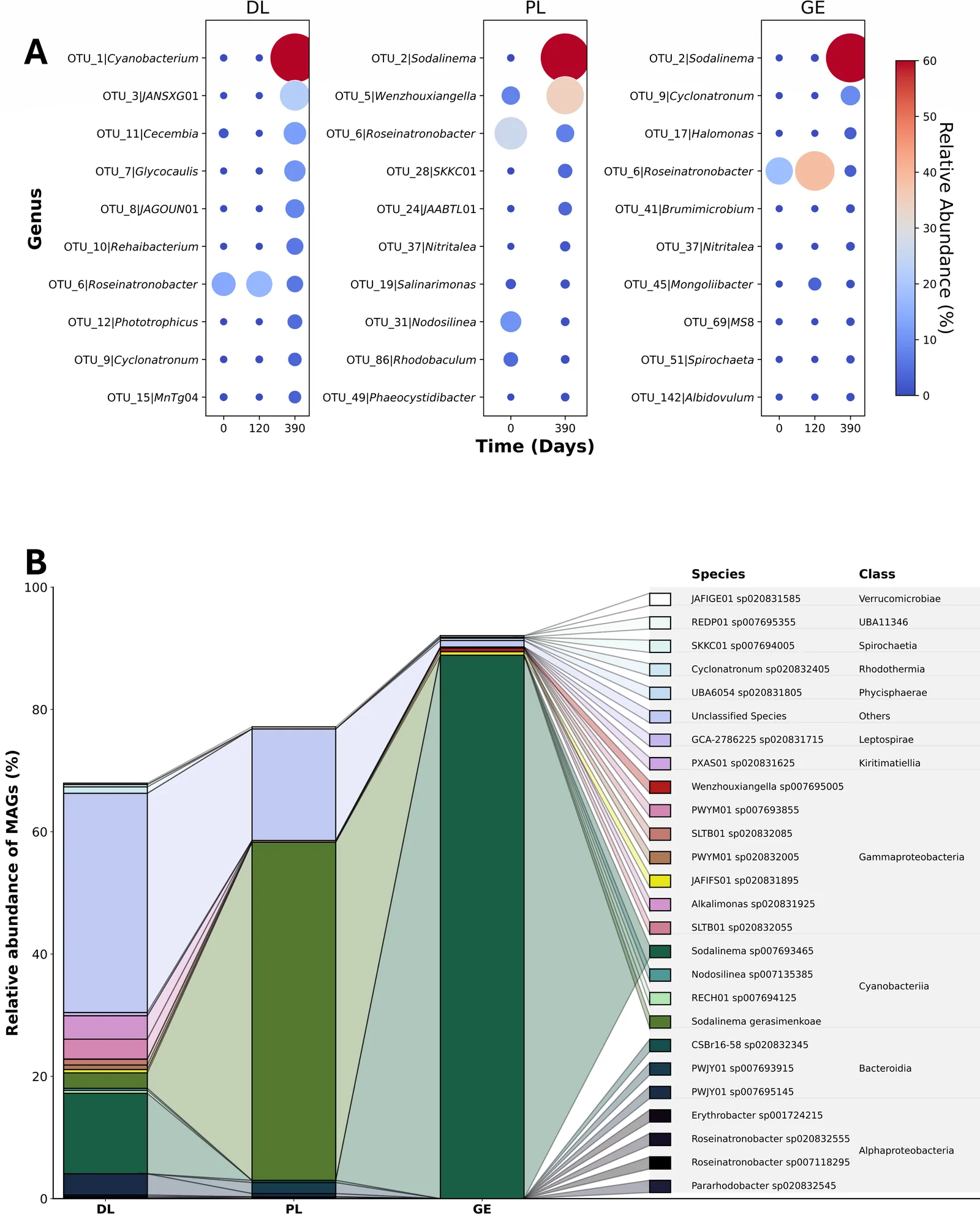
Microbial community dynamics and metagenome-assembled genome (MAG) representation in enriched soda lake consortia. (**A**) Bubble plots showing the temporal shifts in the relative abundance of the ten most abundant genera in each consortium (DL, PL, GE) during 390 days of enrichment. Bubble area and the color correspond to relative abundance (%). (**B**) Relative abundance of MAGs recovered from each consortium after 510 days of enrichment based on mapping of short reads. Colors represent individual MAGs, grouped by taxonomic class. The plot highlights the dominance of *Sodalinema* sp. 007693465 in DL and GE consortia, and *Sodalinema gerasimenkoae* in PL consortium and the distinct heterotrophic assemblages that co-occur with them (Table S7).

The community composition of the enriched consortia was determined by amplicon sequencing (16S and 18S rRNA) at 0, 120, and 390 days after inoculation (Table S2; Fig. 3A). Initially, the mat communities from the different lakes were distinct and yielded different enrichment outcomes (Fig. S1). Alpha diversity metrics (Table S3) showed that DL-T0 exhibited a Shannon diversity index of 3.60, indicating a relatively high level of species richness and evenness. In contrast, while still diverse, the GE-T0 mat started with a slightly lower score of 3.39.

Across all samples, the consortia experienced a consistent decline in diversity over time. Specifically, the DL consortium dropped to 2.53 by day 390, while the GE mat fell to 1.37. These changes indicate that the microbial communities became increasingly dominated by a few specialized species during the enrichment process (Fig. 3A), reducing the overall complexity of the consortia.

Metagenomic sequencing revealed differences in the number of heterotrophic bacterial taxa recovered from each consortium (Tables S4 to S6). Based on the taxonomic classification of metagenome-assembled genome bins, 55 heterotrophic bacterial taxa were identified in the DL consortium and 22 in the PL consortium, both maintained at pH 8.5, compared with 15 in the GE consortium maintained at pH 10.2. These results are consistent with the lower heterotrophic diversity observed under the higher pH condition, as previously reported (22).

Regarding the phototrophic community, the DL consortium had the most taxonomically diverse cyanobacteria after 390 days of cultivation, containing multiple genera, such as *Nodosilinea*, *Cyanobacterium*, *PXDR01*, and *RECH01*. In contrast, the PL and GE consortia contained a single, dominant species of cyanobacteria (*Sodalinema*). For industrial applications, a consortium with a predictable dominant phototrophic member may offer operational advantages by reducing variability in the primary biomass-producing population (37). However, these cultures are not axenic monocultures, and dominance by *Sodalinema* does not eliminate the possibility of invasion or community change. Rather, the observed dominance suggests that *Sodalinema* occupied the phototrophic niche under the enrichment conditions used here.

### Metagenomic reconstruction of cyanobacterial consortia

To explore the potential for secondary metabolite production, short- and long-read sequencing was performed after 510 days of incubation (Fig. 3B). Between days 390 and 510, *Sodalinema* eventually outcompeted *Cyanobacterium*. The higher diversity in DL was consistent with the larger part of the shotgun data that did not assemble into metagenome-assembled genomes (MAGs).

Assembly of the DL consortium yielded 31 high-quality (HQ) (>90% completeness and <10% contamination) MAGs (Table S4), with 16 HQ MAGs for the PL consortium (Table S5) and 5 HQ MAGs for the GE consortium (Table S6). Nine of these high-quality MAGs were cyanobacteria, three of which were successfully closed and circularized.

One of the circularized cyanobacterial MAGs from the DL consortium, measuring 5,049,202 bp, was classified as *Sodalinema*. This MAG’s whole genome sequence contained 4,168 protein-coding genes, resulting in a coding density of 79.4%, which was attributed to the presence of repeat sequences (7.1%). *Sodalinema* MAGs were also recovered from the GE and PL consortia. Consistent with previous results (35), the GC skew plot for the DL *Sodalinema* MAG did not show a conventional symmetrical pattern (Fig. S2).

### Phylogenetic analysis of S*odalinema*

**Fig 4:**
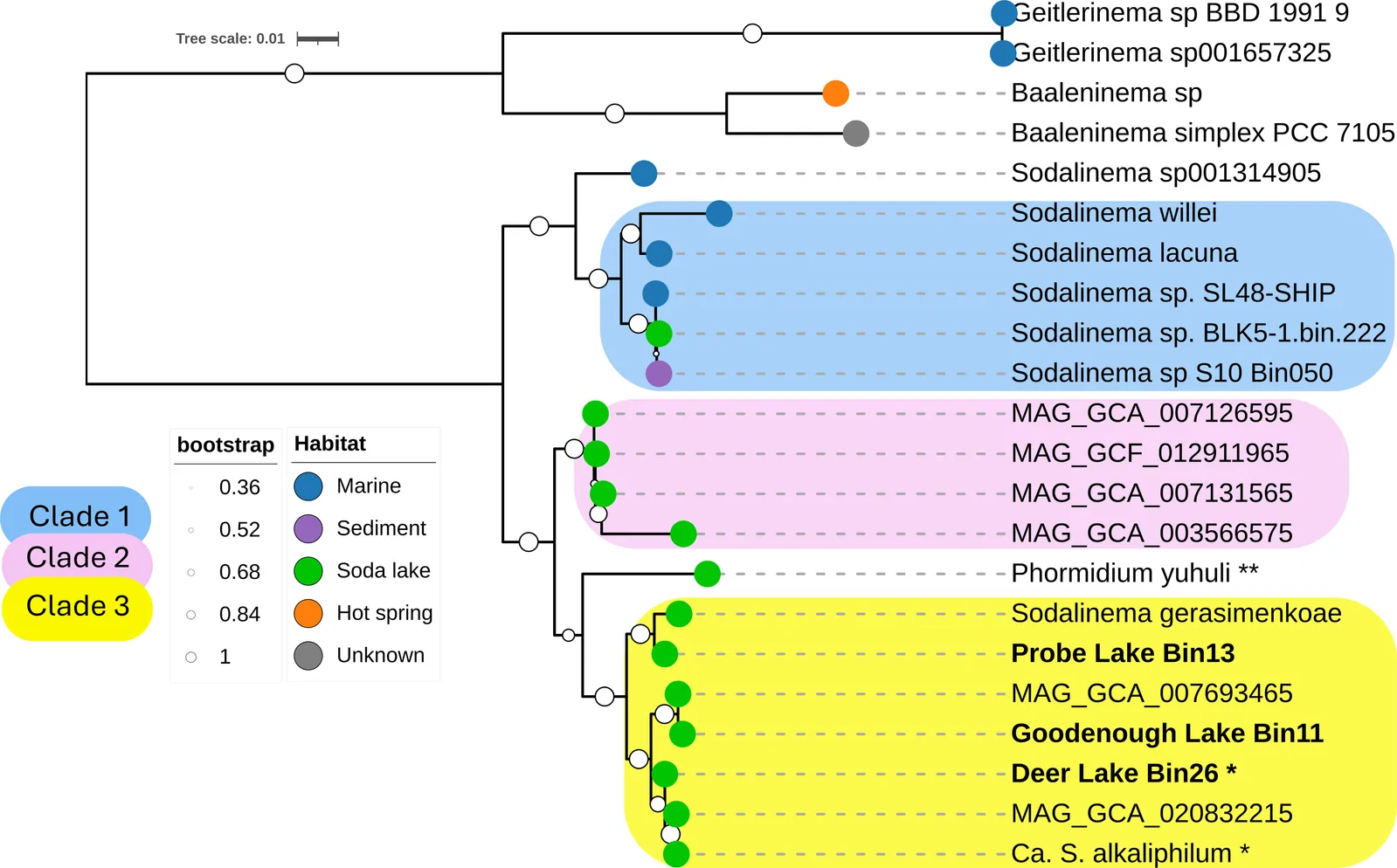
Maximum likelihood phylogenetic tree of *Sodalinema*. A maximum likelihood phylogenetic tree was constructed using a multiple sequence alignment of GTDB conserved marker genes. The tree includes environmental metagenome-assembled genomes (MAGs) and isolate genomes. MAGs created in this study are in bold. *, circularized MAGs; **, isolate with a circularized genome.

The evolutionary relationship of the three new *Sodalinema* MAGs to 15 publicly available *Sodalinema* genomes was investigated phylogenetically based on GTDB-maker genes. The analysis included *Sodalinema* MAGs from Cariboo mats (21, 35), Kulunda Steppe sediments (38–40), and various other habitats (18, 41–45) (Figure 4).

When comparing all *Sodalinema* MAGs generated in this study against previously published MAGs, three distinct clades emerged (see Fig. S3 and Table S1). Clade 1 consisted of *Sodalinema* from marine environments, with ANI values ranging from 93.0% to 99.99%. Clade 2 comprised *Sodalinema* from Russian soda lakes, with ANI values from 98.62% to 99.21%.

Clade 3 contained mainly *Sodalinema* from Canadian lakes, sharing ANI values from 92.1% to 99.9%. With an ANI above 96.4%, the *Sodalinema* enriched from DL and GE consortia represented the same species, whereas *Sodalinema* enriched from the PL consortium was closely related to *Sodalinema gerasimenkoae* (ANI between PL-Bin13 and *S. gerasimenkoae* was 96.4%). In Clade 3, *Sodalinema gerasimenkoae* originated from Russia’s Transbaikal region (45), while the rest of the MAGs originated from Canadian soda lakes from the Cariboo Plateau (21, 35) (Fig. 4).

### Insights into the metabolic capacities of *Sodalinema* consortia

**Fig 5:**
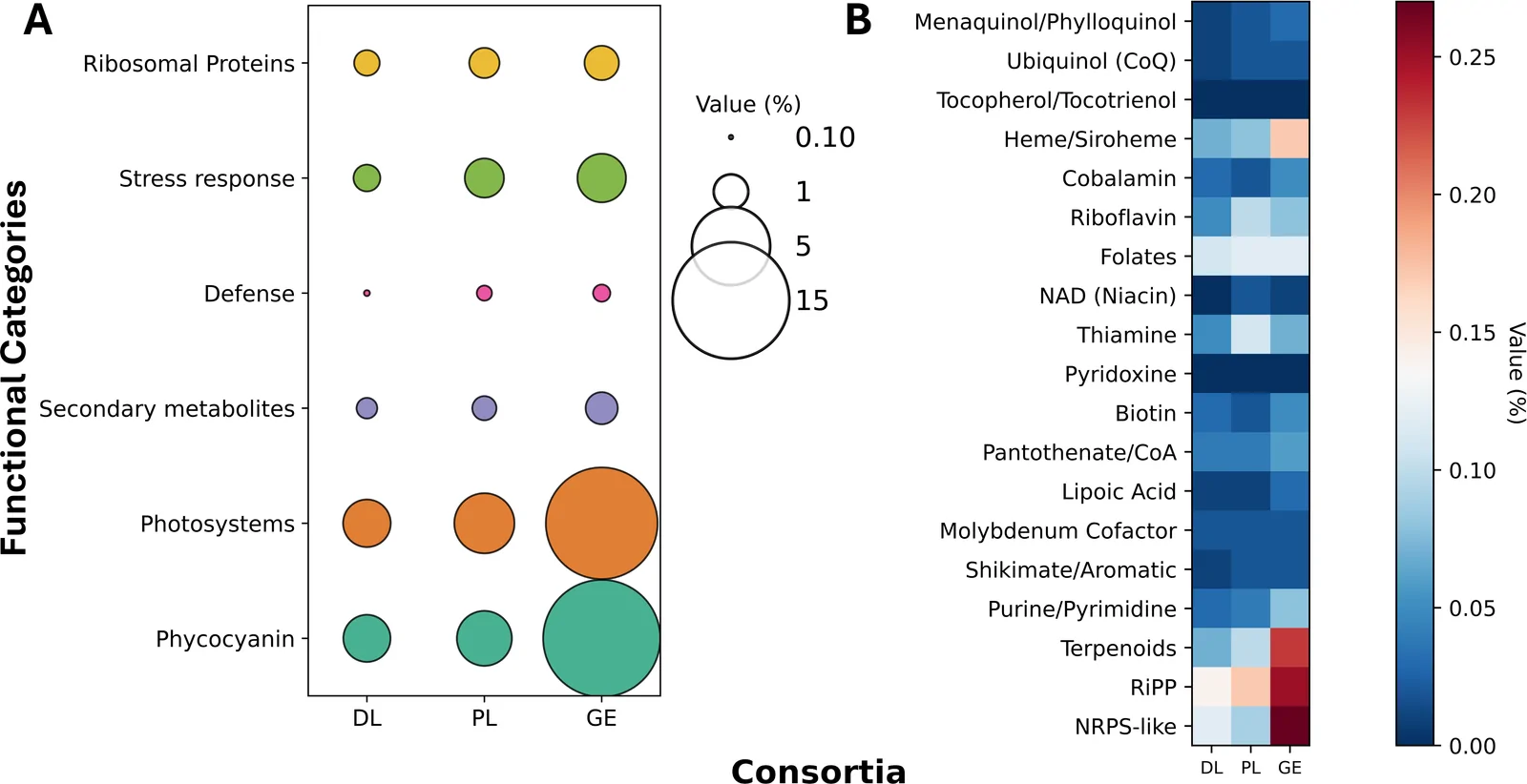
Gene expression analysis of *Sodalinema* metagenome-assembled genomes (MAGs) from three different consortia. (**A**) Gene expression associated with major functional categories. (**B**) Heatmap illustrating the average gene expression of pathways for vitamins, cofactors, and secondary metabolites. The gene expression is shown as a percentage of the transcriptome for each organism (MAG) (Tables S9 and S10).

Microbial mat communities are known to adapt to fluctuations in environmental factors such as light intensity, oxygen, sulfide, pH, and carbon dioxide concentrations (68). To investigate how different *Sodalinema* responded to varying growth conditions, we performed metatranscriptomics for each consortium. RNA was extracted from the consortia at their respective enrichment conditions: Deer Lake (DL) was maintained at pH 8.5, 0.30 M alkalinity, and 21°C; Probe Lake (PL) at pH 8.5, 0.30 M alkalinity, and 30°C; and Goodenough Lake (GE) at pH 10.2, 0.5 M alkalinity, and 21°C. Our analysis revealed the expression of over 10,000 genes across all three *Sodalinema* (Table S8). As expected, gene expression was most active for genes belonging to the photosystem and phycocyanin pathways (Fig. 5A). Notably, *Sodalinema* from the GE consortium (pH 10.2) exhibited a substantially higher relative expression of the genes related to phycocyanin compared with DL and PL (pH 8.5) consortia. The elevated gene expression of phycocyanin within the GE consortium is likely linked to the metabolic requirements of the alkaliphilic environment. This finding is consistent with proteomic data from *Sodalinema* enriched from Goodenough Lake in 2019 (21).

Here, we focused on genes associated with the biosynthesis of secondary metabolites, which often play a crucial role in mediating competitive and adaptive interactions.

*Sodalinema* enriched from Canadian soda lakes also contained a complete pathway for the terpenoid pigments betacarotene and zeaxanthin (see Fig. S4 and Table S11). Genes involved in this pathway made up 0.07, 0.10, and 0.23% of *Sodalinema* transcriptomes in DL, PL, and GE consortia, respectively.

*Sodalinema* MAGs also contained a fragment of a non-ribosomal peptide synthetase (NRPS) (Fig. S5), and a gene cluster potentially encoding a ribosomally synthesized and post-translationally modified peptide (RiPP) (Fig. S6).

We assessed *Sodalinema*’s metabolic self-sufficiency by examining the completeness of core biosynthesis pathways for essential building blocks and cofactors. Pathways for nucleotides (Table S12), including *de novo* purine, adenine, guanine, deoxyribonucleotide, and pyrimidine biosynthesis, were complete. Analysis of amino acid biosynthesis pathways (Table S13) across the three *Sodalinema* MAGs suggested metabolic autonomy.

A complete pathway was identified for biotin (vitamin B_7_) (Fig. S7; Table S14). All core genes of the BioU pathway (KEGG M00950) *bioF* (K00652), *bioU* (K25570), *bioD* (K01935), and *bioB* (K01012) were present. Biotin is a required cofactor for carboxylase enzymes, such as acetyl-CoA carboxylase (46).

A complete biosynthetic pathway was also identified for coenzyme A (CoA) (KEGG M00120). The synthesis of pantothenate (vitamin B_5_) (KEGG M00119), the precursor for CoA, is linked to branched-chain amino acid (BCAA) metabolism. This process involves 3-methyl-2-oxobutanoate hydroxymethyltransferase (*panB*; K00606) and 2-dehydropantoate 2-reductase (*panE*; K00077) to produce pantoate, aspartate 1-decarboxylase (*panD*; K01579) to produce beta-alanine, and pantoate ligase (*panC-cmk*; K13799) for the final condensation. The subsequent CoA biosynthesis pathway (KEGG M00120) proceeds from pantothenate (vitamin B_5_) via four enzymes: pantothenate kinase (*coax*; K03525), phosphopantothenoylcystine decarboxylase (*coaBC*; K13038), pantetheine-phosphate adenylyltransferase (*coaD*; K00954), and dephospho-CoA kinase (*coaE*; K00859).

Analysis of the genomes revealed complete biosynthesis pathways for several other essential cofactors, including NAD/niacin (M00115), lipoic acid (M00881), molybdenum cofactor (M00880), PreQ1 (M00988), and porphyrin/heme (M00121) (Table S14).

In contrast, known biosynthetic pathways for thiamine (B_1_), riboflavin (B_2_), pyridoxine (B_6_), folate (B_9_), cobalamin (B_12_), menaquinone (vitamin K_2_), phylloquinone (vitamin K_1_), and tocopherol (vitamin E) biosynthesis were incomplete in all three *Sodalinema* MAGs (generated in this study). To understand if the enriched *Sodalinema* would be dependent on other consortium members for biosynthesis of these cofactors, we also analyzed these pathways in *Sodalinema yuhuli*—an isolate that can grow autonomously, independent of any other bacteria (47). This comparison showed that *S. yuhuli* also has incomplete pathways for these cofactors (Table S14).

### Metabolic and biosynthetic potential of heterotrophic bacteria

*Roseinatronobacter* MAGs (DL-Bin20 and PL-Bin21) exhibited complete cobalamin (vitamin B_12_) biosynthesis, alongside thiamine (vitamin B_1_) salvage pathways (Table S14).

Heterotrophic consortium members, including *Roseinatronobacter*, *Alkalimonas*, *Kiloniellales,* and *Wenzhouxiangella*, contained biosynthetic gene clusters (BGCs) for various secondary metabolites. *Roseinatronobacter* MAGs (DL-Bin20 and PL-Bin21) encoded the synthesis of N-acyl homoserine lactones (AHLs) (48), which are utilized as quorum-sensing (QS) signaling molecules (49, 50). These MAGs also contained pathways for the osmoprotectants ectoine and N-acetylglutaminylglutamine amide. Additionally, we identified a BGC for betalactone, an NRPS-like antibiotic, and a type 1 polyketide synthase (T1PKS) pathway for prodigiosin, a red pigment with well-documented antimicrobial, algicidal, and anticancer properties (51–53) (Tables S15 to S17; Fig. S8). The identification of such multifunctional secondary metabolites underscores the potential of these alkaliphilic consortia as valuable resources for broader biotechnological and pharmaceutical applications.

*Alkalimonas* MAGs (GE-Bin1 and DL-Bin27) were predicted to produce class I and IV antimicrobial lanthipeptides (lantibiotics) structurally similar to thalassomonsin A/B and andulusicin A/B (54). These ribosomally synthesized and post-translationally modified peptides (RiPPs) (55) showed higher relative gene expression (0.26% and 0.23%, respectively) compared with QS, NRPS/PKS, or terpenoid BGCs (Tables S18 to S20; Fig. S9).

*Kiloniellales* order MAGs (PL-Bin16 and PL-Bin9) (Tables S21 to S23, see Fig. S10) harbored tRNA-dependent cyclodipeptide synthases (CDPSs), suggesting the capacity to synthesize cyclic peptides. Based on homology, this CDPS may produce a compound structurally similar to aspulvinone H/B, a metabolite with antibacterial activity against multidrug-resistant isolates (56). *Kiloniellales* were also predicted to produce hydrogen cyanide (HCN) (57). Three core biosynthetic genes identified in this gene cluster were FAD-binding oxidoreductase, FAD/NAD(P)-binding oxidoreductase, and (2Fe-2S)-binding protein. HncB and HncC each contain typical FAD-NAD binding motifs (58), while HncA contains a cluster of four cysteines, which matches to iron-sulfur binding signatures (59). HNC acts as a powerful respiratory inhibitor utilized for suppressing competing microbes and pathogens (60). Further, a redox-cofactor biosynthetic gene cluster—pyrroloquinoline quinone (PQQ)—was identified. The three core biosynthetic genes were: pyrroloquinoline quinone biosynthesis protein PqqE, pyrroloquinoline quinone biosynthesis peptide chaperone PqqD, and pyrroloquinoline quinone synthase PqqC. The most highly expressed biosynthetic gene cluster was NRPS/PKS with 0.05% and 0.16%, while genes involved in hydrogen cyanide production showed very low gene expression.

*Wenzhouxiangella* MAG (GE-Bin13) was predicted to contain a class II lanthipeptide BGC with the core sequence **C**GMGG**C**QPNPF**S**A**T**L**C**PDGTWII**C**Q, with crosslinked cysteine (**C**) residues shown in bold, Dha, didehydroalanine (**S**), and Dhb, didehydrobutyrine (**T**). Consistent with *Wenzhouxiangella* strain AB-CW3 (61), a bifunctional *lanM* gene responsible for both dehydration and cyclization, was present (62).

## DISCUSSION

This study evaluated the secondary metabolite production potential of *Sodalinema*-dominated cyanobacterial consortia enriched from Canadian alkaline soda lakes. By integrating cultivation, whole-genome sequencing, and metatranscriptomics, we characterized the metabolic architecture of these communities under the enrichment conditions applied to each lake sample. After 510 days, all three enrichment cultures were dominated by *Sodalinema*, despite differences in cultivation pH, alkalinity, and temperature. Because pH, alkalinity, temperature, and lake of origin were not independently varied, this convergence should be interpreted as an observed enrichment outcome rather than evidence that these variables had limited independent effects on community composition. Also, homogenization of the original mat samples combined with the carryover of 50% of the culture during transfer every 15 days may have led to similar outcomes despite application of different enrichment conditions.

Phylogenetic analysis revealed distinct evolutionary trajectories between *Sodalinema* lineages. The high average nucleotide identity (ANI) observed within Clade 2, comprising Russian soda lake isolates, aligns with niche conservatism, where lineages retain ancestral ecological traits within stable environments (63, 64). Conversely, the broader ANI range in Clade 3, which includes isolates from the Cariboo Plateau, suggests diversification driven by the spatial heterogeneity and sharp redox gradients characteristic of microbial mats (65).

Metatranscriptomic data clarified how *Sodalinema* maintains metabolic activity under alkaline stress. At pH 10.2, the organism must sustain internal pH homeostasis against a steep external gradient, requiring significant energy for active transport (66). The observed upregulation of light-harvesting components at high pH likely functions as a compensatory mechanism to supply the ATP and reducing power necessary for both pH regulation and carbon-concentrating mechanisms (67). These transcriptional responses align with previous proteomic observations of *Sodalinema* from Goodenough Lake (21).

The metabolic self-sufficiency of *Sodalinema* is further supported by the presence of complete biosynthetic pathways for essential cofactors. All three MAGs encoded pathways for biotin (B_7_), pantothenate (B_5_), molybdenum cofactor (MoCo), NAD/niacin, lipoic acid, PreQ1, and porphyrin/heme. The expression of the PreQ1 pathway, which produces the hypermodified nucleoside queuosine, suggests a high degree of translational regulation (68, 69). Furthermore, the presence of MoCo biosynthesis supports the function of nitrate reductase and other molybdoenzymes critical for nitrogen assimilation (70), complementing the iron-molybdenum cofactor required for nitrogenase activity (71, 72).

In contrast, several vitamin biosynthetic pathways including thiamine (B_1_), riboflavin (B_2_), pyridoxine (B_6_), folate (B_9_), cobalamin (B_12_), and vitamins K_1_, K_2_, and E, remained incomplete across all *Sodalinema* MAGs. While cyanobacteria are often primary producers, many species exhibit complex vitamin dependencies. For example, despite the importance of thiamine as a cofactor for the Krebs cycle (73), some species rely on external uptake (74, 75). Similarly, while cobalamin and biotin are essential for DNA and lipid synthesis (76), auxotrophy is common in genera, such as *Synechococcus* (77). However, we did not identify expressed vitamin-specific transporters in the *Sodalinema* metatranscriptomes. Thus, vitamin uptake remains a possible explanation for these incomplete pathways, but it was not directly supported by transporter expression in this data set. The similarity in these deficiencies between our MAGs and the axenically grown isolate *Sodalinema yuhuli* suggests that these cofactors are not essential for the genus or that uncharacterized alternative pathways exist. Because the *S. yuhuli* isolate grows successfully in pure culture without heterotrophic partners, a mutualistic supply of these vitamins is apparently not required.

The heterotrophic members of the consortia, including *Kiloniellales*, *Roseinatronobacter*, *Alkalimonas*, and *Wenzhouxiangella*, harbor most of the community’s secondary metabolic potential. These taxa encode diverse biosynthetic gene clusters (BGCs) for non-ribosomal peptide synthetases (NRPSs), polyketide synthases (PKSs), and various ribosomally synthesized and post-translationally modified peptides (RiPPs), such as lanthipeptides and cyclodipeptides. These metabolites likely mediate community dynamics, including quorum sensing via AHLs, niche defense, and redox balancing. The broad distribution of uncharacterized BGCs across these halotolerant and alkaliphilic heterotrophs highlights their potential as reservoirs for natural product discovery, mirroring recent genome-mining efforts that have successfully mapped novel metabolic capabilities in extreme saline-alkaline environments (78). For instance, the redox cofactor PQQ may facilitate the detoxification of reactive oxygen species (53, 60, 79–81). The production of antimicrobials like lanthipeptides and beta-lactones provides a mechanism for competitive exclusion (60, 82), while osmoprotectants, such as ectoine, facilitate coordinated responses to environmental fluctuations (83, 84).

Heterotrophs also appear to supplement the vitamin requirements of the consortia. *Roseinatronobacter* MAGs encode complete cobalamin (B_12_) biosynthesis and thiamine (B_1_) salvage pathways, the latter of which allows for the reclamation of exogenous precursors in nutrient-variable environments. These metabolic contributions suggest that heterotrophs may provide essential cofactors to *Sodalinema*, a symbiotic interaction consistent with reported associations between cyanobacteria and heterotrophic bacteria in other aquatic systems (81, 85–93).

Cyanobacteria are established sources of specialized metabolites with antimicrobial, anticancer, enzyme-inhibitory, photoprotective, and signaling activities (94), and genome mining has expanded access to cryptic biosynthetic pathways that may not be detected under standard cultivation conditions (94). The clinical development of auristatin-based antibody-drug conjugate payloads from the cyanobacterial natural product Dolastatin 10 illustrates the translational potential of cyanobacterial chemistry (95). Beyond the phototroph itself, associated heterotrophs can modulate cyanobacterial physiology and have been incorporated into engineered phototroph–heterotroph systems for light-driven bioproduct formation (96, 97). In our enrichments, heterotrophs encoded most of the detected secondary metabolic potential, including lanthipeptide, beta-lactone, cyclodipeptide, ectoine, carotenoid, and vitamin-associated pathways. Thus, consortium-level analyses can reveal biosynthetic functions that would be missed by focusing on the dominant phototroph alone. However, these annotations indicate biosynthetic potential rather than confirmed metabolite production, and their products, activities, yields, and safety profiles remain unresolved.

The combined capacity of *Sodalinema* to generate primary biomass and heterotrophs to synthesize specialized secondary metabolites suggests potential for integrated bioprocessing applications. In such systems, cyanobacterial biomass could serve as a feedstock for biofuels (98), while heterotrophic BGCs may support the production of higher-value bioproducts such as lanthipeptides, ectoine, and vitamins. These applications remain prospective and require metabolite validation, activity testing, and cultivation optimization. The alkaline adaptation of these consortia may also be useful for cultivation processes where high pH reduces contamination risk and sterilization requirements (99). Overall, this genome-resolved and transcript-resolved analysis provides a framework for evaluating the biosynthetic potential of alkaline cyanobacterial consortia, while highlighting the need for controlled experiments to determine how cultivation conditions, medium exchange regime, and community composition influence metabolite production. This genomic and transcriptomic roadmap provides a foundation for developing alkaline cyanobacterial consortia as microbial platforms for multi-product bioprocessing in extreme environments (93, 100), with potential relevance to circular bioeconomy applications (101).

### Conclusion

Integrating enrichment, cultivation, whole-genome sequencing (WGS), and metatranscriptomics, we characterized three *Sodalinema*-dominated microbial consortia from Canadian soda lakes. Our results confirmed the ecological success of *Sodalinema* in laboratory-based cultivation, demonstrating growth at higher temperatures. Three new *Sodalinema* MAGs belonged to a clade dominated by *Sodalinema* from Canadian soda lakes. Metabolic reconstruction revealed an extensive and actively expressed biosynthetic capacity within the consortia, including pathways for essential cofactors, terpenes, ribosomally synthesized and post-translationally modified peptides (RiPP), and non-ribosomal peptide synthetase (NRPS-like) biosynthesis pathways. Furthermore, the heterotrophic community was shown to augment the system’s capabilities through the contribution of osmoprotectants and secondary metabolites like betalactone, RiPP-like compounds (i.e., lanthipeptides). Our study shows that these microbial communities from extreme environments may be valuable for pharmaceutical and industrial applications. Future work should focus on the biochemical validation of the predicted pathways, which would contribute to biotechnological advances.

## MATERIALS AND METHODS

### Study site and sample collection

Fresh microbial mat samples were obtained from three Canadian alkaline soda lakes: Goodenough (51.33°N, 121.64°W), Deer (51.35°N, 121.24°W), and Probe (51.45°N, 121.39°W) lakes in April 2023, homogenized in HDPE bottles (Nalgene, Thermo Scientific, #B7285), transported to the Microbial Markets laboratory located at the University of Calgary, and stored at room temperature (21°C) in natural light for 18 hours before enrichment.

### Enrichment of cyanobacterial consortia

Selective enrichment of cyanobacterial consortia from microbial mat samples was performed under three cultivation conditions that differed in temperature and growth medium (Table 1). Two consortia were maintained at room temperature (21°C) in 1-L borosilicate glass bottles with continuous stirring at 350–400 rpm. For these enrichments, 50 mL of microbial mat material was inoculated into 1 L of either HA3 or LA3 medium. A third consortium was cultivated at 30°C by inoculating 50 mL of mat material into 200 mL of LA3 medium in an Erlenmeyer flask, with shaking at 135–140 rpm. Cultures were illuminated using full-spectrum LED lights (Model LED24-6400K, SunBlaster, ULC, Langley, BC, Canada) under a 12-h light:12-h dark cycle. Every 15 days, half of the spent medium was replaced with fresh medium.

**TABLE 1:** Medium formulations for selective enrichment of cyanobacterial consortia

Medium	pH	Alkalinity (M)	Na_2_CO_3_ (g/L)	NaHCO_3_ (g/L)
HA1^*a*^	10.2	1	44.7	13.1
HA2^*a*^	10.2	0.33	14.9	4.3
HA3^*a*^	10.2	0.5	22.4	6.5
HA4^*a*^	10.2	0.67	29.8	8.7
LA1^*b*^	8.5	0.48	8.1	27.2
LA2^*b*^	8.5	0.32	5.4	18.2
LA3^*b*^	8.5	0.30	13.6	4.0
LA4^*b*^	8.5	0.16	2.9	9.1
pH1*^a^*^,^^*b*^	8.7	0.5	9.4	26.8
pH2^*a*^^,^^*b*^	9.2	0.5	16.9	15.1
pH3^*a*^^,^^*b*^	9.7	0.5	22.3	6.7
pH4^*a*^^,^^*b*^	10.3	0.5	25	2.5
pH5^*a*^^,^^*b*^	10.9	0.5	26.1	0.7

^*a*^ 0.25 g/L K_2_HPO_4_, 0.26 g/L MgSO_4_, 0.019 g/L CaCl_2_, 0.45 g/L KCl, and 0.34 g/L NaNO_3_ (35).

^*b*^ 0.5 g/L K_2_HPO_4_, 2.5 g/L NaNO_3_, 1 g/L K_2_SO_4_, 1 g/L NaCl, 0.2 g/L MgSO_4_.7H_2_O, and 0.04 g/L CaCl_2_.2H_2_O (102).

Trace metal solution (1 mL/L) (500 mg/L Titriplex III [EDTA], 200 mg/L FeSO_4_.7H_2_O, 10 mg/L ZnSO_4_.7H_2_O, 3 mg/L MnCl_2_.4H_2_O, 30 mg/L H_3_BO_3_, 20 mg/L CoCl_2_.6H_2_O, 1 mg/L CuCl_2_.2H_2_O, 2 mg/L NiCl_2_.6H_2_O, 3 mg /L Na2MoO_4_.2H_2_O), and 16.7 µL/L ferric ammonium citrate (1,596.5 mg/mL) were added after autoclaving of the media. Different nitrogen sources like ammonium chloride (NH_4_Cl) and urea [CO(NH_2_)_2_] were tested to enrich different cyanobacterial consortia. However, these different nitrogen sources did not establish stable cyanobacterial consortia.

### Determination of optimal growth conditions

To measure the growth optimum for temperature (4°C–30°C), pH (8.7–10.9), and alkalinity (0.1–1 M), each cyanobacterial consortium was grown in triplicate using media in Table 1. The pH was measured by a pH meter (Thermo Scientific, VSTAR80) with a pH electrode suitable for alkaline solutions (Fisher Scientific, 8175BNWP). Total consortium growth was estimated by measuring optical density at 750 nm using a Thermo Scientific Evolution 60S spectrophotometer. OD750 was used as a proxy for culture turbidity because this wavelength minimizes direct absorbance from photosynthetic pigments; however, it does not specifically quantify cyanobacterial biomass. The light regime was set to a 12-h light:12-h dark cycle using LED24-6400K lights (SunBlaster, Langley, BC, Canada).

### Amplicon sequencing and data analysis

DNA was extracted from each consortium using the FastDNA Extraction Kit for Soil (MP Biomedicals, Santa Ana, CA, USA, SKU:116560200-CF) after 0, 120 days, and 390 days of selective enrichment, according to the manufacturer’s instructions. DNA concentrations were measured using a Qubit 4.0 fluorometer with dsDNA HS Assay Kit (Thermo Fisher Scientific, Canada). 16S and 18S genes were amplified using universal primer set: 515F-926R (103). Libraries for DNA sequencing were prepared as previously described (20), normalized, and pooled for sequencing on a MiSeq Personal Sequencer (Illumina, San Diego, CA) using the 2 × 300 bp MiSeq Reagent Kit v3. Reads were processed using MetaAmp with the default parameters (104). After merging of paired-end reads (>100  bp overlap and <8 mismatches in the overlapping region), primer trimming, quality filtering (<2 mismatches in primer regions and at most one expected error), and size trimming to 350 bp, reads were clustered into operational taxonomic units (OTUs) of > 97% sequence identity (22). Taxonomic classification of the OTUs was performed using MetaAmp using the arb-silva database (SILVA 138).

### Short-read sequencing and data processing

Genomic DNA was extracted from each consortium using the FastDNA Extraction Kit for Soil (MP Biomedicals, Santa Ana, CA, USA, SKU:116560200-CF) according to the manufacturer’s instructions after 510 days of enrichment. DNA concentrations were measured using a Qubit 4.0 fluorometer with dsDNA HS Assay Kit (Thermo Fisher Scientific, Canada). Libraries were prepared with NEB Ultra II library preparation kit (New England Biolabs, Ipswich, MA) with Covaris shearing to 300–350 bp at the Center for Health Genomics and Informatics in the Cumming School of Medicine, University of Calgary. DNA was sequenced paired-end for 2 × 150 cycles on an Illumina NextSeq 2000 with P1 XLEAP flow cell. BBDuk v38.06 (105) was used with options “qtrim = rl trimq = 15” to trim reads that contained adapter sequences, remove low-quality ends, eliminate contaminants, and filter out reads shorter than 30 bp after cutting the adapters (22).

### High-molecular-weight DNA extraction and long read DNA sequencing

High molecular weight (HMW) DNA was extracted using the Three-Peaks DNA extraction protocol (106) after 510 days. This protocol uses a combination of enzymatic, chemical, and mechanical lysis to extract genomic DNA from the microbial cells without extensive DNA shearing. The DNA concentrations were measured using a Qubit 4.0 fluorometer (Thermo Fisher Scientific, Canada) with a dsDNA HS Assay Kit (Q32851). Before sequencing, size selection (>5 kbp) was performed using the PacBio Short Read Eliminator kit (SRE XS, PacBio, 102-208-200) following the manufacturer’s instructions. Libraries were prepared using the ligation sequencing kit (SQK-LSK114) according to the manufacturer’s instructions. Libraries were each loaded onto a separate MinION R10.4.1 flow cell (Oxford Nanopore Technologies), according to the manufacturer’s instructions. The sequencing run was managed using the MinKNOW v25.03.9 (Oxford Nanopore Technologies) to track and visualize output. Each run progressed for ~ 72 h.

### Long read data processing

Dorado v1.0 was used for offline base-calling and trimming of adapters using the model r10.4.1_e8.2_400bps_sup@v4.3.0 (ONT, https://github.com/nanoporetech/dorado). Chopper v0.10 (107) was used to remove reads shorter than 1000 bases and with quality scores lower than 10. Long-read assembly was performed using MetaMDBG v1.1 (108) with “--in-ont” parameter. Following assembly, the metagenomes were polished with Medaka v2.0.1 (ONT, https://github.com/nanoporetech/medaka) using the “--bacteria” flag. Assemblies were further refined using Polypolish v0.6.0 (109) and Pypolca v0.3.1 (110, 111), which used short-read metagenomic data with the “--careful” parameter. For binning and bin refinement, MetaWRAP v1.3 (112) was used with the “--metabat2 --maxbin2 --concoct” modules. Bin refinement used the completeness and contamination parameters “-c 50 -x 10” to generate metagenome-assembled genomes (MAGs) for subsequent analysis (113). The contiguity of the assembly of these MAGs was assessed using the N50 and genome size metrics provided in the “metawrap_50_10_bins.stats” file. MAGs were taxonomically classified using GTDB-Tk v2.4.0 (114) with reference database version R220. Annotation of the MAGs was performed with MetaErg v2.5.10 (115). The relative abundance of each MAG within the consortia was estimated from both long and short read mapping using the CoverM v0.7.0 genome module (116). GC skew plots were generated with gcskewer (https://github.com/drboothtj/gcskewer) using the “-f” flag. antiSMASH v8.0.2 (117) was used to identify biosynthetic gene clusters. GhostKOALA was used to identify KEGG orthologs (KO) (118), and KEGG Mapper (119) was used to reconstruct metabolic pathways. The biosynthetic gene clusters identified in *Sodalinema* MAGs were compared with the experimentally verified biosynthetic gene clusters using clinker v0.0.31 (120).

### Phylogenetic analysis

Fifteen *Sodalinema* reference genomes, two *Geitlerinema,* and two *Baaleninema* genomes were downloaded from the NCBI. The genomes were selected based on the CheckM2 v1.1.0 (121) completeness > 70% and <10% contamination scores (Table S1). A multiple sequence alignment was created by first extracting the GTDB maker genes using the GTDB-TK v2.4.0 (114) module “gtdbtk identify,” and then aligning them using the module “gtdbtk align.” MEGA12 (122) was used for visualization of the alignment. A maximum likelihood phylogenetic tree was constructed using the LG + G model with 100 bootstraps in MEGA12. The tree was visualized using iTOL v7 (123). Clades were defined based on shared average nucleotide identity (ANI) calculated using fastANI v1.3 using default parameters (124).

### RNA extraction and metatranscriptomic analysis

Total RNA was extracted in triplicate using ZymoBIOMICS DNA/RNA Miniprep Kit (CAT# R2002) following the manufacturer’s instructions, and quantified using a Qubit 4.0 fluorometer (Thermo Fisher Scientific, Canada) with the RNA HS Assay Kit (Q32855). RNA libraries were prepared with a NEB Ultra Directional RNA library Prep instrument using the NEBNext rRNA depletion kit (Bacterial) (New England Biolabs, Ipswich, MA) at the Center for Health Genomics and Informatics, University of Calgary. The final libraries were quantified using the Kapa Library quantification kit for Illumina libraries. RNA libraries were sequenced paired-end for 2 × 100 cycles on a NovaSeq S2 instrument. Raw read quality was analyzed using FastQC v12.1 (125) and multiQC v1.29 (126). Raw reads were filtered using “bbduk.sh” with “qtrim = rl trimq = 15 minlength-30,” and the reads were mapped to the features generated MAGs using the “seal.sh” script with “ambig = random” from BBTools (105). To calculate transcript-per-million values (TPM), transcripts mapped to each genomic feature were first divided by the feature length in kilobases to get reads per kilobase (RPK). The raw read counts from “seal.sh” were used to calculate RPK by dividing the reads by length of the gene and multiplied by 1000. Subsequently, all RPK values were summed and divided by a factor of 1,000,000 to get a “scaling factor.” Finally, RPK values for each feature were divided by the “scaling factor” to get TPM values. TPM values were normalized to each MAG by dividing the TPM values of each feature by the sum of TPM values of all features of the MAG. MMseqs2 v17.b804f (127) was used to cluster the protein sequences with “--min-seq-id 0.98,” to enable comparison of gene expression between nearly identical MAGs present in different consortia.

### Microscopy

Microscopy was performed using an upright Axio Imager.A2 (Zeiss, Germany) fitted with 10×, 40×, and 63× objectives and using direct interference contrast. Grayscale images were captured using the Axiocam 506 mono camera (Zeiss, Germany) with ZEN 2012 blue edition software (Zeiss, Germany).

## Data Availability

All data sets generated during the current study are available in ENA under PRJEB108561. The 16S rRNA amplicon sequence Biosamples are SAMEA121652307–SAMEA121652314. The Biosamples for the metagenome-assembled genomes (MAGs) are SAMEA122999517–SAMEA122999618. The Biosamples for the raw shotgun metagenomic reads are SAMEA121652417–SAMEA121652419. The Biosamples for the raw RNA-seq reads are SAMEA123009599–SAMEA123009607. All codes used to generate the plot are available at https://github.com/Ruchita-0310/Cyanobacterial_consortia_NP and https://github.com/Ruchita-0310/Metatranscriptomics.

## SUPPLEMENTAL MATERIAL

Supplemental figures aem.00780-26-s0001.docx Fig. S1 to S10.

Supplemental tables aem.00780-26-s0002.xlsx Tables S1 to S23.

ASM does not own the copyrights to Supplemental Material that may be linked to, or accessed through, an article. The authors have granted ASM a non-exclusive, world-wide license to publish the Supplemental Material files. Please contact the corresponding author directly for reuse.
