# Supplementary material for: Genome mining of alkaliphilic cyanobacterial consortia: identification of biosynthetic gene clusters in Sodalinema and associated heterotrophs
Source: Appl Environ Microbiol. 2026 Sep 8;92(10):e00780-26. doi: 10.1128/aem.00780-26 (PMC13688837; doi:10.1128/aem.00780-26)
Supplement: Supplementary file 1 [file aem.00780-26-s0001.docx]

**Supplementary information**

**Genome mining potential of alkaliphilic cyanobacteria: Identification of biosynthetic gene clusters in *Sodalinema* and associated heterotrophs**

Authors: Ruchita Solanki*^1^, Everet Wiszniak^2^, Lianchun Yi^1^, Gamaliel Cabria^1^, Marc Strous^1^, Francisco Daniel Davila Aleman^2^

Author Affiliations:

^1^ Department of Earth, Energy, and Environment, University of Calgary, Calgary, Alberta, Canada

^2^ Department of Biological Sciences, University of Calgary, Calgary, Alberta, Canada

*Corresponding Author

Email address: [ruchita.solanki@ucalgary.ca](mailto:ruchita.solanki@ucalgary.ca)

**List of Contents**

**Supplementary Figures**

Supplementary Figure 1 - Beta-diversity of microbial communities across sites and timepoints

Supplementary Figure 2 - Analysis of GC Skew and Cumulative GC Skew of circularized *Sodalinema* MAG (Bin26) enriched from Deer Lake consortium

Supplementary Figure 3 - Average Nucleotide Identity (ANI) Matrix of *Sodalinema* Genomes

Supplementary Figure 4 - Complete Pathway for Zeaxanthin Biosynthesis (C40 Terpenoid Biosynthesis) identified in *Sodalinema* MAGs generated in this study

Supplementary Figure 5 - Comparative analysis of Non-ribosomal peptide synthetase (NRPS) - like biosynthetic gene cluster.

Supplementary Figure 6 - Comparative genomic analysis of Ribosomally synthesized and Post-translationally modified Peptide (RiPP) biosynthetic gene cluster.

Supplementary Figure 7 - Biotin Biosynthesis Pathway (BioU Pathway - KEGG M00950) in *Sodalinema* MAGs generated in this study

Supplementary Figure 8 - Expression profiles of secondary metabolite and cofactor biosynthetic genes in *Rosienatronobacter* MAGs

Supplementary Figure 9 - Expression profiles of secondary metabolite and cofactor biosynthetic genes in *Alkalimonas* MAGs.

Supplementary Figure 10 - Expression profiles of secondary metabolite and cofactor biosynthetic genes in *Kiloniellales* MAGs.

**Figure S1: Beta-diversity of microbial communities across sites and timepoints.** Non-metric Multidimensional Scaling (NMDS) plot based on (Supplementary Table S3) dissimilarities of microbial community compositions. Points are colored by sampling site (DL in orange, GE in light blue, and PL in green) and shaped by sampling timepoint (T0 = circles, T120 = triangles, and T390 = crossed squares). The stress value is 0.02.

**Figure S2: Analysis of GC Skew and Cumulative GC Skew of circularized *Sodalinema* MAG (Bin26) enriched from Deer Lake consortium.** This consortium was enriched at 8.5 pH, 0.3M alkalinity, and room temperature (21°C).

**Figure S3: Average Nucleotide Identity (ANI) Matrix of *Sodalinema* Genomes.** This heatmap represents the pairwise Average Nucleotide Identity (ANI) values, expressed as a percentage, calculated between 15 publicly available *Sodalinema* genomes from different habitats. The color intensity corresponds to the ANI percentage, with deep red indicating high identity and deep blue indicating low identity. *Sodalinema* MAGs generated in this study are in bold.

**Figure S4: Complete Pathway for Zeaxanthin Biosynthesis (C40 Terpenoid Biosynthesis) identified in *Sodalinema* MAGs generated in this study.** This diagram illustrates the multi-stage biochemical pathway leading to the synthesis of the xanthophyll pigment, Zeaxanthin (Supplementary Table S10). Stage 1: MEP Pathway (Non-Mevalonate / KEGG:M00364): The pathway begins with precursors Pyruvate and G3P (Glycolytic intermediates) which generate the C5 precursors IPP (Isopentenyl Diphosphate) and DMAPP (Dimethylallyl Diphosphate). These combine to form GGPP (Geranylgeranyl pyrophosphate), a C20 compound. Stage 2: C40 Scaffold Synthesis (Poly-cis intermediate route): Two molecules of GGPP are condensed by 15-cis-Phytoene Synthase (CRTB, K02593) to form 15-cis-Phytoene (C40). Stage 3: Zeaxanthin Biosynthesis (KEGG:M01045): Lycopene is cyclized by Lycopene Cyclase (CRT B/Y, K14605, K14606) to produce Beta-Carotene. Finally, Beta-Carotene Hydroxylase (CRT Z, K00504) acts on Beta-Carotene to form the final product, Zeaxanthin, a cyclic xanthophyll.

**Figure S5: Comparative analysis of Non-ribosomal peptide synthetase (NRPS) - like biosynthetic gene cluster.**

Synteny plot illustrating the conservation and gene organization of NRPS-like cluster across *Photorhabdus luminescens* subsp. *laumondii* TTO1 and three *Sodalinema* metagenome-assembled genomes (MAGs) (Bin11, Bin26, and Bin13) generated from this study. Bin11 was enriched from GE consortium, Bin26 from DL consortium, and Bin13 from PL consortium. NRPS gene cluster in *Photorhabdus luminescens* subsp. *laumondii* TTO1 was experimentally verified.

**Figure S6: Comparative genomic analysis of Ribosomally synthesized and Post-translationally modified Peptide (RiPP) biosynthetic gene cluster.**

Synteny plot illustrating the conservation and gene organization of RiPP gene cluster across *Nostoc*, *Bacillus*, *Halalkalibacterium*, and one *Sodalinema* metagenome-assembled genome (MAG) (Bin11) generated from this study. Bin11 was enriched from GE consortium. RiPP gene cluster in *Nostoc,* *Bacillus*, and *Halalkalibacterium*  were experimentally verified.

**Figure S7:** **Biotin Biosynthesis Pathway (BioU Pathway - KEGG M00950) in *Sodalinema* MAGs generated in this study.** This figure outlines the biochemical steps for the final stages of Biotin (Vitamin B7) synthesis, starting from the precursor Pimeloyl-CoA. The pathway involves the sequential transformation of Pimeloyl-CoA through three key intermediates: Pimeloyl-CoA is converted to 8-Amino-7-oxononanoate by 8-Amino-7-oxononanoate Synthase (BioF, K00582). 8-Amino-7-oxononanoate is converted to 7,8-Diaminononanoate. The specific enzyme for this step is often associated with the BioU Family Protein (BioU, K03570). 7,8-Diaminononanoate is then converted to Dethiobiotin by Dethiobiotin Synthase (BioD, K01935). Finally, Dethiobiotin is converted to the final product, Biotin (Vitamin B7), by Biotin Synthase (BioB, K01932) (Supplementary Table S14).

**Figure S8: Expression profiles of secondary metabolite and cofactor biosynthetic genes in *Rosienatronobacter* MAGs.** Heatmap illustrating the relative expression levels (%) of genes associated with various metabolic pathways across two MAGs, Bin20 (DL consotium) and Bin21 (PL consotium). DL-Bin20 was enriched at 8.5 pH, 0.3M alkalinity, and room temperature (21°C). PL-Bin21 was enriched at 8.5 pH, 0.3M alkalinity, and 30°C.

**Figure S9: Expression profiles of secondary metabolite and cofactor biosynthetic genes in *Alkalimonas* MAGs.** Heatmap illustrating the relative expression levels (%) of genes associated with various metabolic pathways across two MAGs, Bin1 (GE consotium) and Bin27 (DL consotium). GE-Bin1 was enriched at 10.2 pH, 0.5M alkalinity, and room temperature (21°C). DL-Bin27 was enriched at 8.5 pH, 0.3M alkalinity, and room temperature (21°C).

**Figure S10: Expression profiles of secondary metabolite and cofactor biosynthetic genes in *Kiloniellales* MAGs.** Heatmap illustrating the relative expression levels (%) of genes associated with various metabolic pathways across two MAGs, Bin9 and Bin16 from PL consortium. PL consortium was enriched 8.5 pH, 0.3M alkalinity, and 30°C.
